# Validity assessment of oral health promotion activities targeting the older population for community care in South Korea: A Delphi study

**DOI:** 10.1111/ger.12768

**Published:** 2024-07-10

**Authors:** Jin‐Sun Choi, Soo‐Myoung Bae, Bo‐Mi Shin, Hyo‐Jin Lee, Hye‐Young Yoon, Sun‐Jung Shin

**Affiliations:** ^1^ Department of Dental Hygiene, College of Dentistry Gangneung‐Wonju National University Gangneung‐si South Korea; ^2^ Research Institute of Oral Science Gangneung‐Wonju National University Gangneung‐si South Korea; ^3^ Research Institute of Dental Hygiene Science Gangneung‐Wonju National University Gangneung‐si South Korea

**Keywords:** community care, Delphi surveys, elderly, South Korea

## Abstract

**Objective:**

The purpose of this study was to establish an oral health activity assessment tool for older people and evaluate its validity.

**Background:**

To provide reasonable and efficient oral health promotion services with limited medical resources, a tool including categories and items of oral health promotion activities for older people should be prepared.

**Materials and methods:**

The tool initially consisted of 76 items on oral health promotion activities for older people classified into assessment‐performance‐evaluation stages. Topics for each stage included general and oral health, daily health, oral health status, behaviour, and awareness. In addition, two Delphi surveys were conducted on 10 experts who met the selection criteria, and the final items were derived based on the review opinions.

**Results:**

As a result of the first and second Delphi surveys, the content validity for all items was ≥0.60 and the content validity index was ≥0.80. In the first survey, the degree of convergence in some items was 0‐0.88. After modifying the contents according to expert opinions, the degree of convergence was improved from 0 to 0.50 in the second survey. The degree of agreement ranged from 0.75 to 1.00, indicating that experts agreed. Finally, a total of 65 items were derived.

**Conclusion:**

A 65‐item tool was derived through two Delphi surveys for the assessment of oral health activities for older people. The use of the tool developed in this study would likely contribute to better prevention of oral diseases and the promotion of oral health among older people.

## 1 INTRODUCTION

In South Korea (hereafter Korea), the proportion of the older population is rapidly increasing owing to the country's low fertility rate and increased average life expectancy. Among the Organization for Economic Cooperation and Development (OECD) countries, the fastest population aging is occurring in Korea,^1^ and it is forecasted to become a super‐aged society by 2025, with individuals aged ≥65 years accounting for over 20% of the entire population.^2^ Moreover, the total medical expenditure under the Korean national health insurance system has been increasing by an average of 9.3% each year since 2014, while the medical expenditure for the older population has been increasing by an average of 12.6%, showing that medical expenditure for the older population is also on the rise.^3^ Accordingly, the Ministry of Health and Welfare of Korea has implemented “community health care,” a type of community‐based integrated care service, to relieve the increasing burden of medical expenditure for the older population, indicating a shift in the community‐based welfare paradigm.

The community care system in Korea is defined as a community‐led social service policy that promotes integrated support for housing, health/medical care, nursing care, care, and independent living so residents can live together as a community while enjoying services that meet their individual needs.^4^ Other countries that are experiencing population aging, including the UK and Japan, have also invested effort in establishing a community care system with health services for the older population to use in their daily life.

Korea's community care policy was introduced in 2019 as a pilot program with eight local governments, building the foundation to provide community care by 2023‐2025, and making community care universally available after 2026.^5^ In particular, to address the tremendous increase in medical expenditure for the older population, resulting from Korea's emergence as a super‐aged society, it is necessary to focus more on intensive home healthcare service and preventive care for chronic diseases, which are key challenges in healthcare and medical care.

Systemic and chronic diseases are common among the older population and are also closely associated with oral diseases. A drug prescribed for treating a chronic disease may have adverse effects on oral health,^6^ while poor oral health can exacerbate systemic diseases.^7^ Michaud et al^8^ demonstrated that periodontal disease is deeply associated with the development of respiratory diseases, cardiovascular diseases, and cancer; additionally, tooth loss is associated with gastritis and duodenal ulcers. Similarly, other studies have reported that oral health is mutually associated with cardiovascular, cerebrovascular, and respiratory diseases and diabetes mellitus.^9^, ^10^ According to the 2019 National Health Statistics, the mastication difficulty and limited oral function rates among Koreans aged ≥65 years were 36.9% and 39.8%, respectively, indicating that more than one‐third of the older population has an uncomfortable experience with limited oral function. Meanwhile, only 56.6% of the older population has 20 or more natural teeth, indicating that the oral health status of the older population remains poor.^11^ When the oral health behaviours and status of the older population in applicable regions prior to the home visit program were investigated through the community care pilot program, the results revealed low levels of toothbrushing practice and dentist visits, and the number of natural teeth and dental plaque index were also low.^12^

According to previous studies, while tools exist for identifying individuals at risk of oral frailty and functional impairment or for measuring oral health‐related quality of life,^13^, ^14^, ^15^, ^16^ those that can be used in the implementation of policies such as the community care system for assessing oral health, applying appropriate intervention according to the condition, and subsequently evaluating the contents, are lacking. To establish reasonable and efficient public oral health promotion with limited healthcare resources, it is necessary to prepare a tool that includes specific classifications and items of oral health promotion activities based on objective evidence. Accordingly, this study aimed to assess the validity of items that can be used in the community care policy for assessing, intervening in (implementation), and evaluating the oral health of the older population.

## 2 METHODS

### 2.1 Development of research tool

This study was conducted with ethical approval from the Institutional Review Board (IRB) of G University (IRB No: GWNUIRB‐2022‐19). In this study, a total of 76 items were used, including 72 items on oral health promotion activities for the older population used by Song,^17^ which were revised and supplemented, and four items that were newly added. The 76 items were classified into assessment (n = 52), implementation (n = 19), and evaluation (n = 5) stages. The assessment stage included the subcategories of systemic health (systemic condition, subjective symptoms, physiological function, medical history, systemic diseases, infectious diseases, motor function, and physical characteristics), oral health (oral status, physiological function, motor function, subjective symptoms, oral disease symptoms, and defective prostheses), and daily health (communication, vitality, lifestyle habits, dietary habits, family composition, and local environment). The implementation stage included the subcategories of oral health, diet, and others. Finally, the evaluation stage included the subcategories of oral health status, oral health behaviour, oral health awareness, and satisfaction.

The questionnaire items were evaluated on a 5‐point Likert scale (1‐5 points) for content validity and specialisation, degree of difficulty, and importance, as proposed by Kim et al,^18^ Chae et al,^19^ and Chi et al,^20^ respectively. In addition, the questionnaire for the first Delphi survey was developed to include close‐ended questions for assessing the content validity of the oral health promotion activities that had been devised for the older population and open‐ended questions that could be used by the expert panel to draft opinions on revised and supplemented items (Table 1).

**TABLE 1:** Validity assessment scale.

Category	Question	Scale
Content validity	Is this item suitable as an oral health promotion activity for the older population?	Likert scale (1‐5 points)
Clarity	Is this oral health promotion activity for the older population written clearly with understandable terms?	Likert scale (1‐5 points)
Degree of difficulty	What is the degree of difficulty of this oral health promotion activity for the older population?	Likert scale (1‐5 points)
Importance	How important is this oral health promotion activity for the older population?	Likert scale (1‐5 points)
Specialisation	How specialised is this oral health promotion activity for the older population?	Likert scale (1‐5 points)
Subjectivity	Who would be the appropriate subject of this oral health promotion activity for the older population?	#1: Dental hygienist #2: Dental hygienist + care worker

### 2.2 Delphi survey

#### 2.2.1 Selection of participants

The expert panel for the Delphi survey comprised 10 members, and the inclusion criteria were as follows: licensed dentists or dental hygienists with experience in oral health‐related research for the older population (including oral health‐related projects for the older population) and lecturers or experts with at least 5 years of clinical dentistry experience who consented to participate in the study. The selected experts participated in two rounds of questionnaire surveys over a period of approximately 1 month from the date of IRB approval. All participants responded to both rounds of the Delphi survey. The participants held licences as dentists or dental hygienists, and their professions included dentists, dental hygiene faculty, and public officials engaged in oral health‐related activities. The mean age of the participants was 47.1 ± 7.7 years (range: 30‐59 years), mean educational experience was 14.9 ± 6.3 years (range: 4‐23 years), and mean clinical experience was 15.4 ± 11.4 years (range: 2‐33 years).

#### 2.2.2 Delphi surveys

In the first Delphi survey, content validity, clarity, specialisation, degree of difficulty, importance, and subjectivity were investigated, and validation was performed by reviewing the first Delphi survey results and revising, combining, and deleting items. The participants were informed in advance about the study, and consent was obtained from each participant. Subsequently, the questionnaire was sent to the participants via email.

After revising the contents in accordance with the first survey results, the second Delphi survey was performed. In the second survey, validation was performed on the oral health promotion activity assessment, implementation, and evaluation items that were revised and supplemented based on the first survey results. Validity was determined using the same methods used in the first Delphi survey result analysis.

#### 2.2.3 Data analysis

From the collected data, the results were derived using MS Office Excel. To objectively validate the contents regarding oral health promotion activities, the first Delphi survey results were used to calculate the content validity ratio (CVR), CVI, coefficient of variation (CV), degree of convergence, and degree of agreement, as proposed by Kwak.^21^ The criteria for each validity indicator were determined based on the participation of a panel of 10 experts, with a minimum acceptable value of 0.62 for CVR and 0.78 for CVI. CV was judged to be stable when it was less than 0.5; 0.5‐0.8 was relatively stable; and 0.8 or more was unstable. Convergent validity was judged to be higher as the value approached zero, while discriminant validity was considered higher as the value approached one.

## 3 RESULTS

In the first Delphi survey, 71 of the 76 items regarding oral health activities showed a CVI value ≥0.80 and 72 items showed a CVR value ≥0.60. The degree of agreement scores ranged between 0.50 and 1.00 points, while the degree of convergence scores ranged between 0.00 and 0.88 points. The mean clarity scores ranged between 3.80 and 5.00 points, the mean degree of difficulty scores ranged between 2.30 and 5.00 points, and the mean specialisation scores ranged between 2.90 and 5.00 points (Table S1).

The assessment, implementation, and evaluation items for oral health activities were revised and supplemented by considering the contents of the subjective expert evaluation. Among the 52 assessment items for oral health activities, the experts deemed eight items unsuitable for the assessment. Accordingly, these eight items, which included assessment of facial expression, expression ability, physical condition (subjective symptoms), aging, gait speed, severity of physical paralysis, and local environment (local characteristics and health and welfare support system), were deleted. Among the implementation items, one item related to bed and wheelchair mobility was deleted. Among the evaluation items, the evaluation item for bleeding on probing (BOP) before and after implementation was deleted. Moreover, to clarify the meaning of the terminology used in five assessment items, four implementation items, and two evaluation items, the subjects and predicates were revised to supplement the content. Consequently, of the 76 items, 10 were deleted and 11 were revised and supplemented, resulting in a total of 66 items (Table S1).

In the second Delphi survey, all items except one showed a CVR value ≥0.60 and CVI value ≥0.80. The scores for degree of convergence were 0.00‐0.88 points in the first survey, but after the content was revised based on the expert opinions, the scores improved to 0.00‐0.50 points in the second survey. The scores for degree of agreement were 0.75‐1.00 points, indicating that expert consensus was reached. The mean scores for clarity of each item were 3.80‐4.40 in the first survey and 4.40‐5.00 points in the second survey, indicating that clarity of each item was assured. The second survey results showed that the content validity improved from the first survey, and after deleting one item that did not meet the cut‐off validity score, a total of 65 items remained (Table S2).

Of the 66 items, five or more of the 10 experts selected 41 items (62%) that could be assessed by dental hygienists. There were oral paralysis, chewing difficulty, eating disorder/dysphagia, discomfort due to reduced salivation, dry mouth, oral muscle atrophy, difficulty with mouth opening and closing, tongue function, current oral symptoms, oral disease, tooth loss status, lichen planus/candidiasis, gingival recession, alveolar bone resorption, dental attrition, tooth wear, defective restoration, unfitness of restoration, denture breakage, and wearing/removing dentures. For those items to check systemic conditions, the experts were of the view that care workers and dental hygienists could conduct the assessment. In the implementation stage, the items that dental hygienists would assess were oral health education, customised toothbrushing, treatment of stomatitis and intraoral wounds, professional dental plaque control, teeth cleaning, fluoride application, denture care, treatment for xerostomia, eating/chewing training, dietary assessment, and provision of oral health information. Cleaning the inside of the mouth, mouth rinsing, oral callisthenics, first aid, communication, and dietary counselling were items that care workers and dental hygienists could assess. In the evaluation stage, items regarding evaluation of BOP, O'Leary index for remaining teeth, oral health care behaviours (frequency of toothbrushing, interdental care, etc.), changes in oral health knowledge and awareness before and after implementation, and program satisfaction were items for which dental hygienists could assess (Table S2).

## 4 DISCUSSION

There is a dearth of objective tools for measuring all components of systemic and oral health. For community care‐based oral health care services for the older population, the tool developed and evaluated in this study will likely provide more efficient health care services using limited resources. Informed by two rounds of expert Delphi surveys, the final tool comprised 65 validated items. Items relating to oral health promotion activities were categorised into assessment, implementation, and evaluation stages.

The development of a tool that comprises items to record the general and oral health status of older people and inform customised oral health management is a strength of this study. The study also has some limitations, including the lack of verification of the effectiveness, convenience and suitability of use in community health programs, and not considering the characteristic of the care environment. Nevertheless, the oral health activity items developed in this study could be used in a screening tool to develop appropriate interventions for older people.

In the assessment stage, items that should be considered for implementing oral healthcare service, such as communication, mobility, subjective symptoms, posture maintenance, mouth opening/closing function, usual dietary behaviours, and usual vitality, were ultimately included and validated. Items such as communication, mobility, physical fitness, weight loss, nutrition, and mental health are highly associated with tooth loss, and hence, they are important factors,^22^ while basic data identifying medical history, anthropometric measures, memory, and nutritional status can serve as evidence for choosing the right intervention according to the health status of the older population.^23^ Using a scale for measuring the ability to perform daily living activities can be helpful for identifying health problems the older population may have and providing healthcare services.^24^

By contrast, eight items for assessing the older individual's facial expression, expression ability, physical condition (subjective symptoms), aging, gait speed, physical paralysis, and local environment (local characteristics, health and welfare support system, etc.) were deleted. Of these items, “Assess the older individual's local environment (local characteristics, health, and welfare support system, etc.)” was deleted because the content validity score in the first Delphi survey did not meet the cut‐off. Nevertheless, these items should be considered as a factor that can influence the health and oral health of the older population because older individuals typically spend more time in the community they live in owing to reduced physical and cognitive abilities, which (in turn) restrict their social activities and limited mobility due to reduced physical and cognitive abilities, They may also be influenced more by community environments than other populations.^25^ In addition, the creation of a healthy environments and socioeconomic factors must be supported, as they are key factors needed to ensure welfare improvement, better quality of life, and better health of the older population.^26^ To provide healthcare service customised for each older individual based on community care, the characteristics of that community and information about the health and welfare support system should also be considered.

The 19 items in the implementation stage comprised those related to oral health education, mouth cleaning and rinsing, toothbrushing, treatment for stomatitis, dental plaque control, teeth cleaning, dental caries control, denture care, treatment for xerostomia, oral callisthenics, swallowing function training, dietary counselling, and assessment, body position changes appropriate for oral health care, first aid, communication, and provision of oral health care information. The item regarding bed and wheelchair mobility, which did not meet the cut‐off CVI value, and the item regarding body position change appropriate for oral health care, which was determined to be unnecessary, were deleted. However, with respect to the deleted item “Implement bed and wheelchair mobility,” body position change, bed mobility, and wheelchair mobility are considered important in basic nursing knowledge and the legal care worker development process.^27^ As oral home health care may be provided for older individuals at nursing facilities, homes, and nursing hospitals, education should also be considered for safe and proper bed and wheelchair mobility.

Among the five evaluation stage items, the item for the evaluation of BOP before and after implementation was deleted, and the item for the evaluation of oral health status before and after implementation, PHP index was changed to O'Leary index. Some experts mentioned that the selection of the assessment index for the oral status of older individuals should consider the possibility of the person having few remaining teeth. Accordingly, the evaluation stage ultimately included items regarding dental plaque index, oral hygiene practice, changes in knowledge and awareness, and program satisfaction.

The consensus view was that dental hygienists would be the most likely discipline to assess and implement items on the evaluation of oral and peri‐oral tissues, oral hygiene care, oral care treatment, and dietary counselling. Accordingly, the items deemed to require the expertise of dental hygienists were confirmed to be 41 of the 66 items (approximately 62%). In the Korean care system for the older population, the proportion of dental treatment and oral care for older individuals' oral health is low or non‐existent. The Korean Elderly Long‐term Care Insurance System was adopted in July 2008, and implemented as a “social insurance system that provides services to support the physical activities and daily living activities of people who have difficulty performing them on their own owing to advanced age or geriatric conditions with the purpose of ensuring stability in later life and lessening the burden on their family.”^28^ Eligible individuals may be admitted to a long‐term care facility or receive home visit services for physical activities, household activities, and nursing, depending on the care classification criteria. In long‐term care facilities that provide such services, care workers and nurses are primarily responsible for providing care. According to the Long Term Care Insurance Statistical Yearbook, the number of nurses, nursing assistants, and care workers has been increasing each year; there were 3645 nurses, 14 196 nursing assistants, and 507 473 care workers as of 2021.^29^ However, as of 2021, only 12 dental hygienists in the entire country were working in long‐term care facilities; moreover, all were working in home care and none were working in an actual care facility.^29^

In the “Community‐based integrated care system” of Japan, a country that has already entered the era of population aging, dental hygienists affiliated with local medical institutions provide information about oral care products and oral dietary intake.^30^ In the process of dental and general hospitals collaborating within the community, dental hygienists work together with ward nurses to suggest oral care methods that are appropriate for the oral status of each patient.^31^ In Japanese long‐term care facilities for older individuals, dental hygienists play an important role in improving the quality of life of residents by providing individualised oral care to all residents and recommending dental treatment to those who need it.^32^ Further, Hopcraft et al^33^ recommended increasing the utilisation of dental hygienists for the efficient use of resources and for ensuring the safety of the residents in care facilities for older individuals, since dental hygienists have the necessary knowledge for performing oral examinations or planning oral hygiene treatment. To increase the utilisation of dental hygienists in nursing facilities and ensure their long‐term employment, it is essential to establish an appropriate fee for dental hygiene services, thereby enhancing their role as professional personnel.^34^

Policies are needed to expand the role of dental hygienists in the field of oral rehabilitation, which can contribute to improvement in oral health associated with systemic diseases, as Korea prepares for a super‐aged society. Moreover, a collaborative body comprising different occupational groups should be integrated into the community care‐based healthcare structure, so that the structure enables continued collaboration to create a positive cycle of health and oral health care for the older population.

The experts in this study suggested that care workers would likely be able to use the tool to assess and implement items that require relatively little processional knowledge about oral health, such as those that check systemic conditions. In Korea, the Welfare of Senior Citizens Act stipulates that “a person who establishes and operates a welfare institution for senior citizens shall employ caregivers who perform duties such as providing support for physical activities or household activities of senior citizens, etc., as prescribed by Ordinance of the Ministry of Health and Welfare,”^35^ and based on these legal grounds, care workers who play a role in providing support for daily living activities, assistance with meals and personal hygiene, assistance with medication administration, and support for physical activities are utilised in the care process for older individuals.

There are 507 473 care workers currently working in the field, showing an increase of 12.5% from 2020 to 2021.^29^ Although the legally mandated curriculum for care workers includes oral care education regarding “personal hygiene and environmental care protection,” oral care is included only in a very small section within “assisting cleanliness of mouth, hair, hands/feet, and perineal region,” while specialised education content that must actually be used is non‐existent.^36^ Moreover, the standard textbook for care workers provided by the Ministry of Health and Welfare only contains content for assisting with oral hygiene, and there is no education content on the specialised knowledge needed for periodontal disease and xerostomia, which older individuals often experience.^37^ According to Jung et al,^38^ care workers who provide care services to residents of long‐term care facilities for older individuals lack oral health‐related knowledge, and they recognise the need for oral health care education. Similarly, Charadram et al^39^ reported that oral health care‐related education and training are essential for professionals who provide care to the older population. Therefore, clinical practice guidelines or education programs including high‐quality oral health care education should be developed in the curriculum for care workers who will provide care services to the older population in care facilities, and oral health specialists should be dispatched to the field through policy support.^1^

The community integrated care policy of Korea, which is in its pilot stage, has been criticised for its lack of “interconnectedness and inseparability,” “comprehensiveness,” and “multi‐stakeholder partnerships.”^40^ In Japan, for convalescent rehabilitation of older individuals in the field of oral rehabilitation, care is provided to patients through a team‐based care system, in which many specialists from various occupational groups, including physicians, nurses, nutritionists, physical therapists, occupational therapists, speech therapists, and dental hygienists, are assigned to a single patient, while the team also addresses any oral health problems.^41^ Accordingly, Korea should also provide team‐based care with members included from various occupational groups for the care and rehabilitation of older individuals. Moreover, it is necessary to expand the role of dental hygienists by securing dental and oral care to promote oral health, which is closely associated with systemic health.

In addition to its use at an individual or community level, the findings of this study could also be used to inform national policies on the health and oral health of the older Korean population, and likely in other countries. In the future, to establish the introduction of the field of oral health in Korea's community care, opportunities should be provided to examine projects in the oral field currently being piloted, analyse policies, and reflect positive overseas cases in policies.^30^, ^31^, ^32^

## 5 CONCLUSION

In this study, 65 items for an oral health activity tool were obtained and validated. If the tool is applied to community care models or oral health promotion programs for the older population and oral health care consisting of a standardised model is continuously provided, then such efforts can contribute to oral disease prevention and oral health promotion in the older population. Furthermore, expanding the role of dental hygienists to include dental and oral care, which are closely linked to overall health, and providing multidisciplinary medical care within elderly care and rehabilitation systems is crucial.

## AUTHOR CONTRIBUTIONS

J‐S Choi secured research funding and contributed to research design, survey implementation, statistical analysis, interpretation of research results, manuscript writing, and final manuscript review. S‐J Shin contributed to research design, development of survey tools, interpretation of research results, manuscript writing, and final manuscript review. S‐M Bae contributed to research design and manuscript writing. B‐M Shin contributed to research design, interpretation of research results, and manuscript writing. H‐J Lee contributed to the creation of survey tools, interpretation of research results, and manuscript writing. H‐Y Yoon contributed to research design and manuscript writing. All authors reviewed and approved the submitted version of the manuscript.

## FUNDING INFORMATION

This work was supported by a grant from the National Research Foundation of Korea, which is funded by the Korea government (MSIT; No. 2021R1G1A1094141).

## CONFLICT OF INTEREST STATEMENT

The authors declare that there are no conflicts of interest related to this study.

## ETHICAL APPROVAL

The study was conducted in accordance with the Declaration of Helsinki, and approved by the Institutional Review Board (or Ethics Committee) of the Institutional Review Board of G University (IRB No: GWNUIRB‐2022‐19).

## Supporting information


Tables S1‐S2

## Data Availability

The data that support the findings of this study are available from the corresponding author, S‐J Shin, upon reasonable request.
