# Supplementary material for: Validity assessment of oral health promotion activities targeting the older population for community care in South Korea: A Delphi study
Source: Gerodontology. 2024 Jul 10;42(1):93–9. doi: 10.1111/ger.12768 (PMC11870635; doi:10.1111/ger.12768)
Supplement: Supplementary file 1 — Tables S1‐S2 [file GER-42-93-s001.docx]

**Supplementary tables**

Table S1. First expert Delphi survey results

| Category | | Item | Content validity | | | | | | Specialization | Resulting action |
| --- | --- | --- | --- | --- | --- | --- | --- | --- | --- | --- |
|  |  |  | Mean±SD | CVR^*^ | CVI^**^ | CV^***^ | Degree of convergence | Degree of agreement | Mean±SD |  |
| **Part 1. Assessment stage** |  |  |  |  |  |  |  |  |  |  |
| 1.1. Systemic health |  |  |  |  |  |  |  |  |  |  |
| Systemic condition | 1) | Body type (systemic condition) | 4.2±0.8 | 0.60 | 0.80 | 0.19 | 0.50 | 0.75 | 3.2±0.8 | Revised^†^ |
|  | 2) | Facial expression. | 3.5±0.9 | 0.00 | 0.50 | 0.24 | 0.50 | 0.71 | 2.9±0.9 | Deleted^††^ |
|  | 3) | Severity of physical paralysis | 4.4±0.8 | 0.60 | 0.80 | 0.19 | 0.50 | 0.80 | 3.5±1.1 | Revised^†^ |
| Subjective symptoms | 4) | Current symptoms (subjective symptoms) | 4.4±0.8 | 0.60 | 0.80 | 0.19 | 0.50 | 0.80 | 3.8±1.1 | Revised^†^ |
|  | 5) | Physical condition (subjective symptoms) | 4.5±0.7 | 0.80 | 0.90 | 0.16 | 0.50 | 0.80 | 3.7±1.1 | Deleted^††^ |
|  | 6) | Aging | 4.2±1.0 | 0.60 | 0.80 | 0.25 | 0.50 | 0.78 | 3.5±1.1 | Deleted^††^ |
| Physiological function | 7) | Auditory and visual functions | 4.6±0.7 | 0.80 | 0.90 | 0.15 | 0.38 | 0.85 | 3.4±2.0 |  |
|  | 8) | Vital signs | 4.8±0.4 | 1.00 | 1.00 | 0.09 | 0.00 | 1.00 | 4.3±1.1 |  |
|  | 9) | Dietary behavior | 4.7±0.5 | 1.00 | 1.00 | 0.10 | 0.38 | 0.85 | 3.6±0.8 |  |
| Medical history and systemic diseases | 10) | Treatment status for systemic diseases | 4.8±0.4 | 1.00 | 1.00 | 0.09 | 0.00 | 1.00 | 4.1±0.9 |  |
|  | 11) | Medication status for systemic diseases | 4.7±0.5 | 1.00 | 1.00 | 0.10 | 0.38 | 0.85 | 4.0±0.9 |  |
|  | 12) | Previous and current medical history | 4.8±0.4 | 1.00 | 1.00 | 0.09 | 0.00 | 1.00 | 4.1±0.9 |  |
| Infectious diseases | 13) | Infectious diseases (HBV, HCV, HIV, tuberculosis, etc.) according to systemic condition | 4.4±0.8 | 0.60 | 0.80 | 0.19 | 0.50 | 0.80 | 4.3±1.1 | Revised^†^ |
| Motor function | 14) | Gait speed | 4.0±0.8 | 0.40 | 0.70 | 0.20 | 0.75 | 0.63 | 3.2±0.8 | Deleted^††^ |
|  | 15) | Severity of physical paralysis | 4.4±0.8 | 0.60 | 0.80 | 0.19 | 0.50 | 0.80 | 4.0±0.8 | Deleted^††^ |
|  | 16) | Ability to maintain posture | 4.4±0.8 | 0.60 | 0.80 | 0.19 | 0.50 | 0.80 | 3.6±1.1 |  |
| Physical characteristics | 17) | Musculoskeletal status | 4.6±0.7 | 0.80 | 0.90 | 0.15 | 0.38 | 0.85 | 4.1±0.9 |  |
|  | 18) | Respiratory status | 4.6±0.7 | 0.80 | 0.90 | 0.15 | 0.38 | 0.85 | 4.0±1.2 |  |
|  | 19) | Digestive status | 4.6±0.7 | 0.80 | 0.90 | 0.15 | 0.38 | 0.85 | 4.2±0.9 |  |
| 1.2. Oral health |  |  |  |  |  |  |  |  |  |  |
| Oral health needs | 20) | The desire to receive oral care services | 4.8±0.4 | 1.00 | 1.00 | 0.09 | 0.00 | 1.00 | 2.9±0.9 |  |
| Oral status | 21) | Oral paralysis | 4.7±0.7 | 0.80 | 0.90 | 0.14 | 0.00 | 1.00 | 4.4±0.8 |  |
|  | 22) | Chewing difficulty | 4.9±0.3 | 1.00 | 1.00 | 0.06 | 0.00 | 1.00 | 4.1±0.7 |  |
|  | 23) | Eating disorders and dysphagia | 5.0±0.0 | 1.00 | 1.00 | 0.00 | 0.00 | 1.00 | 4.1±0.9 |  |
| Physiological function | 24) | Discomfort due to decreased salivation | 4.9±0.3 | 1.00 | 1.00 | 0.06 | 0.00 | 1.00 | 3.8±0.9 |  |
|  | 25) | Severity of dry mouth | 4.9±0.3 | 1.00 | 1.00 | 0.06 | 0.00 | 1.00 | 4.5±0.9 |  |
|  | 26) | Severity of oral muscle atrophy | 4.7±0.5 | 1.00 | 1.00 | 0.10 | 0.38 | 0.85 | 4.9±0.3 |  |
| Motor function | 27) | Difficulty with mouth opening and closing | 4.8±0.4 | 1.00 | 1.00 | 0.09 | 0.00 | 1.00 | 4.5±0.9 |  |
|  | 28) | Tongue function | 4.8±0.6 | 0.80 | 0.90 | 0.13 | 0.00 | 1.00 | 4.4±1.1 |  |
| Oral status | 29) | Current oral symptoms | 4.9±0.3 | 1.00 | 1.00 | 0.06 | 0.00 | 1.00 | 4.5±0.7 |  |
|  | 30) | Oral diseases | 5.0±0.0 | 1.00 | 1.00 | 0.00 | 0.00 | 1.00 | 4.6±0.7 | Revised^†^ |
|  | 31) | Oral hygiene status | 4.8±0.6 | 0.80 | 0.90 | 0.13 | 0.00 | 1.00 | 4.4±0.7 |  |
|  | 32) | Tooth loss status | 4.8±0.6 | 0.80 | 0.90 | 0.13 | 0.00 | 1.00 | 4.2±0.8 |  |
| Oral disease symptoms | 33) | Periodontal disease | 5.0±0.0 | 1.00 | 1.00 | 0.00 | 0.00 | 1.00 | 5.0±0.0 | Revised^†^ |
|  | 34) | Lichen planus | 4.7±0.5 | 1.00 | 1.00 | 0.10 | 0.38 | 0.85 | 4.9±0.3 | Revised^†^ |
|  | 35) | Candidiasis | 4.8±0.4 | 1.00 | 1.00 | 0.09 | 0.00 | 1.00 | 4.8±0.4 | Deleted^††^ |
|  | 36) | Gingival recession status | 4.7±0.5 | 1.00 | 1.00 | 0.10 | 0.38 | 0.85 | 4.0±1.3 |  |
| Oral status | 37) | Bone resorption status | 4.4±1.0 | 0.80 | 0.90 | 0.22 | 0.50 | 0.80 | 4.3±1.3 | Revised^†^ |
|  | 38) | Dental attrition status | 4.4±0.8 | 0.60 | 0.80 | 0.19 | 0.50 | 0.80 | 4.2±1.0 |  |
|  | 39) | Tooth wear status | 4.4±0.8 | 0.60 | 0.80 | 0.19 | 0.50 | 0.80 | 4.3±0.8 |  |
| Defective prosthesis | 40) | Unfitness of defective restoration | 4.9±0.3 | 1.00 | 1.00 | 0.06 | 0.00 | 1.00 | 4.8±0.4 | Revised^†^ |
|  | 41) | Breakage of defective restoration | 4.9±0.3 | 1.00 | 1.00 | 0.06 | 0.00 | 1.00 | 4.7±0.5 | Revised^†^ |
|  | 42) | Unfitness of dentures | 4.9±0.3 | 1.00 | 1.00 | 0.06 | 0.00 | 1.00 | 4.9±0.3 |  |
|  | 43) | Denture breakage | 4.9±0.3 | 1.00 | 1.00 | 0.06 | 0.00 | 1.00 | 4.6±0.7 |  |
|  | 44) | Denture wearing and removing status | 5.0±0.0 | 1.00 | 1.00 | 0.00 | 0.00 | 1.00 | 4.3±1.1 | Revised^†^ |
| Communication | 45) | Expression ability | 3.6±0.8 | 0.20 | 0.60 | 0.23 | 0.50 | 0.75 | 3.4±0.5 | Deleted^††^ |
|  | 46) | Communication ability | 4.5±0.7 | 0.80 | 0.90 | 0.16 | 0.50 | 0.80 | 3.4±0.5 | Revised^†^ |
| Vitality | 47) | Vitality (will to live: interest in things) | 4.4±0.7 | 0.80 | 0.90 | 0.16 | 0.50 | 0.78 | 3.3±0.7 |  |
| 1.3. Daily health |  |  |  |  |  |  |  |  |  |  |
| Psychological problems | 48) | Psychological problems (depression, bipolar disorder, social isolation, etc.) | 4.3±0.7 | 0.80 | 0.90 | 0.16 | 0.50 | 0.75 | 3.9±0.7 |  |
| Lifestyle habits | 49) | Daily lifestyle habits (eating behaviors, lifestyle behaviors, hobbies, etc.) | 4.4±0.7 | 0.80 | 0.90 | 0.16 | 0.50 | 0.78 | 3.6±0.5 |  |
| Dietary habits | 50) | Dietary habits (nutritional intake status, diet, etc.) | 4.6±0.7 | 0.80 | 0.90 | 0.15 | 0.38 | 0.85 | 3.3±0.8 |  |
| Family composition | 51) | Family composition (living arrangement, family history) | 4.2±0.8 | 0.60 | 0.80 | 0.19 | 0.50 | 0.75 | 2.7±0.8 |  |
| Local environment | 52) | Local environment (local characteristics, health and welfare support system, etc.) | 3.9±0.9 | 0.20 | 0.60 | 0.22 | 0.88 | 0.56 | 3.6±0.8 | Deleted^††^ |
| **Part 2. Implementation stage** |  |  |  |  |  |  |  |  |  |  |
| 2.1. Oral health |  |  |  |  |  |  |  |  |  |  |
|  | 53) | Oral health education for oral care methods that suit the characteristics of the older individual for the prevention of oral diseases | 5.0±0.0 | 1.00 | 1.00 | 0.00 | 0.00 | 1.00 | 4.8±0.4 |  |
|  | 54) | Clean the inside of the older individual’s mouth using a sponge brush or gauze swab | 5.0±0.0 | 1.00 | 1.00 | 0.00 | 0.00 | 1.00 | 3.6±0.8 |  |
|  | 55) | Rinse the inside of the older individual’s mouth using mouthwash and physiological saline | 4.9±0.3 | 1.00 | 1.00 | 0.06 | 0.00 | 1.00 | 3.5±0.9 |  |
|  | 56) | Professional toothbrushing customized for the older individual | 4.9±0.3 | 1.00 | 1.00 | 0.06 | 0.00 | 1.00 | 4.2±0.8 | Revised^†^ |
|  | 57) | Treat the older individual’s stomatitis and intraoral wounds | 4.8±0.4 | 1.00 | 1.00 | 0.09 | 0.00 | 1.00 | 4.3±0.8 |  |
|  | 58) | Professional dental plaque control (Watanabe) on the older individual | 4.8±0.6 | 0.80 | 0.90 | 0.13 | 0.00 | 1.00 | 4.9±0.3 |  |
|  | 59) | Teeth cleaning (scaling, polishing, etc.) on the older individual | 4.7±0.7 | 0.80 | 0.90 | 0.14 | 0.00 | 1.00 | 4.9±0.3 |  |
|  | 60) | Fluoride application on the older individual for caries control | 4.8±0.6 | 0.80 | 0.90 | 0.13 | 0.00 | 1.00 | 4.8±0.4 | Revised^†^ |
|  | 61) | Denture care (cleaning, storage, oral care after removal) for the older individual | 5.0±0.0 | 1.00 | 1.00 | 0.00 | 0.00 | 1.00 | 3.8±0.9 |  |
|  | 62) | Treatment for xerostomia (application of lip balm, oral moisturizer, etc.) on the older individual | 4.3±0.8 | 0.60 | 0.80 | 0.19 | 0.50 | 0.78 | 4.0±0.8 | Revised^†^ |
|  | 63) | Intra- and extra-oral massage (including oral calisthenics) on the older individual to prevent xerostomia | 4.5±0.9 | 0.60 | 0.80 | 0.19 | 0.38 | 0.85 | 4.2±0.9 |  |
| 2.2. Nutrition and diet |  |  |  |  |  |  |  |  |  |  |
|  | 64) | Eating and swallowing function training to improve the older individual’s oral functions | 4.6±0.7 | 0.80 | 0.90 | 0.15 | 0.38 | 0.85 | 4.6±0.7 |  |
|  | 65) | Dietary counseling to maintain the older individual’s nutritional status | 4.5±0.7 | 0.80 | 0.90 | 0.16 | 0.50 | 0.80 | 4.1±0.9 |  |
|  | 66) | Dietary assessment to maintain the older individual’s nutritional status | 4.4±0.7 | 0.80 | 0.90 | 0.16 | 0.50 | 0.78 | 4.1±0.9 |  |
| 2.3. Others |  |  |  |  |  |  |  |  |  |  |
|  | 67) | Implement body position change for the older individual’s oral health care. | 4.3±1.1 | 0.60 | 0.80 | 0.25 | 0.50 | 0.80 | 3.4±1.0 | Revised^†^ |
|  | 68) | Implement bed and wheelchair mobility. | 3.7±1.1 | 0.00 | 0.50 | 0.29 | 0.88 | 0.50 | 3.0±0.7 | Deleted^††^ |
|  | 69) | Implement first aid and basic life support in case of emergency situation. | 4.5±0.7 | 0.80 | 0.90 | 0.16 | 0.50 | 0.80 | 4.6±0.7 |  |
|  | 70) | Communicate with consideration for the older individual’s systemic diseases and psychological characteristics. | 4.4±0.8 | 0.60 | 0.80 | 0.19 | 0.50 | 0.80 | 4.3±0.8 |  |
|  | 71) | Provide oral health care- and promotion-related information (oral health education, how to link to/utilize local healthcare services, etc.) to older individuals/guardians. | 4.9±0.3 | 1.00 | 1.00 | 0.06 | 0.00 | 1.00 | 3.9±1.0 |  |
| **Part 3. Evaluation stage** |  |  |  |  |  |  |  |  |  |  |
| Oral health status | 72) | Oral health status before and after implementation: Evaluate BOP | 4.7±0.7 | 0.80 | 0.90 | 0.14 | 0.00 | 1.00 | 4.9±0.3 | Deleted^††^ |
|  | 73) | Oral health status before and after implementation: Evaluate O’Leary index for remaining teeth | 4.5±0.9 | 0.60 | 0.80 | 0.19 | 0.38 | 0.85 | 5.0±0.0 | Revised^†^ |
| Oral health behaviors | 74) | Oral health care behaviors before and after implementation (frequency of toothbrushing, interdental care, etc.) | 5.0±0.0 | 1.00 | 1.00 | 0.00 | 0.00 | 1.00 | 3.9±0.9 |  |
| Oral health awareness | 75) | Changes in oral health knowledge and awareness before and after implementation | 4.5±0.7 | 0.80 | 0.90 | 0.16 | 0.50 | 0.80 | 3.9±0.7 | Revised^†^ |
| Satisfaction | 76) | Participant’s satisfaction with the program (reuse intention, recommendation intention, program satisfaction) | 5.0±0.0 | 1.00 | 1.00 | 0.00 | 0.00 | 1.00 | 3.3±1.2 |  |

^*^CVR: content validity rati

^**^CVI: content validity index

^***^CV: coefficient of variation

^†^Revised: Revised based on the opinions of the first expert panel and an integrated discussion among researchers.

^††^Deleted: Deleted due to ineligibility based on the validity criteria of the first-round content.

Table S2. Second expert Delphi survey results

| Category | | Item | Content validity | | | | | | Subjectivity | |
| --- | --- | --- | --- | --- | --- | --- | --- | --- | --- | --- |
|  |  |  | Mean±SD | CVR^*^ | CVI^**^ | CV^***^ | Degree of convergence | Degree of agreement | Dental hygienist | Dental hygienist +  care worker |
| **Part 1. Assessment stage** |  |  |  |  |  |  |  |  | N=10 | N=10 |
| 1.1. Systemic health |  |  |  |  |  |  |  |  |  |  |
| Systemic condition | 1) | Body type (systemic condition) | 3.7±1.0 | 0.20 | 0.60 | 0.26 | 0.50 | 0.75 | 0 | 10 |
|  | 2) | Facial expression. | - | - | - | - | - | - | 0 | 0 |
|  | 3) | Severity of physical paralysis | 4.5±0.7 | 0.80 | 0.90 | 0.16 | 0.50 | 0.80 | 2 | 8 |
| Subjective symptoms | 4) | Current symptoms (subjective symptoms) | 4.5±0.9 | 0.60 | 0.80 | 0.19 | 0.38 | 0.85 | 1 | 9 |
|  | 5) | Physical condition (subjective symptoms) | - | - | - | - | - | - | 0 | 0 |
|  | 6) | Aging | - | - | - | - | - | - | 0 | 0 |
| Physiological function | 7) | Auditory and visual functions | 4.7±0.7 | 0.80 | 0.90 | 0.14 | 0.00 | 1.00 | 0 | 10 |
|  | 8) | Vital signs | 4.9±0.3 | 1.00 | 1.00 | 0.06 | 0.00 | 1.00 | 2 | 8 |
|  | 9) | Dietary behavior | 4.8±0.4 | 1.00 | 1.00 | 0.09 | 0.00 | 1.00 | 0 | 10 |
| Medical history and systemic diseases | 10) | Treatment status for systemic diseases | 5.0±0.0 | 1.00 | 1.00 | 0.00 | 0.00 | 1.00 | 2 | 8 |
|  | 11) | Medication status for systemic diseases | 4.9±0.3 | 1.00 | 1.00 | 0.06 | 0.00 | 1.00 | 2 | 8 |
|  | 12) | Previous and current medical history | 5.0±0.0 | 1.00 | 1.00 | 0.00 | 0.00 | 1.00 | 2 | 8 |
| Infectious diseases | 13) | Infectious diseases (HBV, HCV, HIV, tuberculosis, etc.) according to systemic condition | 4.7±0.5 | 1.00 | 1.00 | 0.10 | 0.38 | 0.85 | 2 | 8 |
| Motor function | 14) | Gait speed | - | - | - | - | - | - | 0 | 0 |
|  | 15) | Severity of physical paralysis | - | - | - | - | - | - | 0 | 0 |
|  | 16) | Ability to maintain posture | 4.3±1.1 | 0.60 | 0.80 | 0.25 | 0.50 | 0.80 | 0 | 10 |
| Physical characteristics | 17) | Musculoskeletal status | 4.4±0.8 | 0.60 | 0.80 | 0.19 | 0.50 | 0.80 | 0 | 10 |
|  | 18) | Respiratory status | 4.6±0.7 | 0.80 | 0.90 | 0.15 | 0.38 | 0.85 | 2 | 8 |
|  | 19) | Digestive status | 4.5±0.7 | 0.80 | 0.90 | 0.16 | 0.50 | 0.80 | 1 | 9 |
| 1.2. Oral health |  |  |  |  |  |  |  |  |  |  |
| Oral health needs | 20) | The desire to receive oral care services | 4.9±0.3 | 1.00 | 1.00 | 0.06 | 0.00 | 1.00 | 2 | 8 |
| Oral status | 21) | Oral paralysis | 4.9±0.3 | 1.00 | 1.00 | 0.06 | 0.00 | 1.00 | 6 | 4 |
|  | 22) | Chewing difficulty | 5.0±0.0 | 1.00 | 1.00 | 0.00 | 0.00 | 1.00 | 5 | 5 |
|  | 23) | Eating disorders and dysphagia | 5.0±0.0 | 1.00 | 1.00 | 0.00 | 0.00 | 1.00 | 5 | 5 |
| Physiological function | 24) | Discomfort due to decreased salivation | 4.9±0.3 | 1.00 | 1.00 | 0.06 | 0.00 | 1.00 | 5 | 5 |
|  | 25) | Severity of dry mouth | 4.9±0.3 | 1.00 | 1.00 | 0.06 | 0.00 | 1.00 | 9 | 1 |
|  | 26) | Severity of oral muscle atrophy | 4.6±0.5 | 1.00 | 1.00 | 0.11 | 0.50 | 0.80 | 10 | 0 |
| Motor function | 27) | Difficulty with mouth opening and closing | 4.8±0.4 | 1.00 | 1.00 | 0.09 | 0.00 | 1.00 | 8 | 2 |
|  | 28) | Tongue function | 4.8±0.6 | 0.80 | 0.90 | 0.13 | 0.00 | 1.00 | 9 | 1 |
| Subjective symptoms | 29) | Current oral symptoms | 4.9±0.3 | 1.00 | 1.00 | 0.06 | 0.00 | 1.00 | 8 | 2 |
|  | 30) | Oral diseases | 5.0±0.0 | 1.00 | 1.00 | 0.00 | 0.00 | 1.00 | 10 | 0 |
|  | 31) | Oral hygiene status | 4.8±0.6 | 0.80 | 0.90 | 0.13 | 0.00 | 1.00 | 8 | 2 |
| Oral disease symptoms | 32) | Tooth loss status | 4.8±0.6 | 0.80 | 0.90 | 0.13 | 0.00 | 1.00 | 8 | 2 |
|  | 33) | Periodontal disease | 5.0±0.0 | 1.00 | 1.00 | 0.00 | 0.00 | 1.00 | 10 | 0 |
|  | 34) | Lichen planus | 4.8±0.4 | 1.00 | 1.00 | 0.09 | 0.00 | 1.00 | 10 | 0 |
|  | 35) | Candidiasis | - | - | - | - | - | - | 0 | 0 |
| Oral status | 36) | Gingival recession status | 4.8±0.4 | 1.00 | 1.00 | 0.09 | 0.00 | 1.00 | 10 | 0 |
|  | 37) | Bone resorption status | 4.4±0.7 | 0.80 | 0.90 | 0.16 | 0.50 | 0.78 | 10 | 0 |
|  | 38) | Dental attrition status | 4.2±1.0 | 0.60 | 0.80 | 0.25 | 0.50 | 0.78 | 9 | 1 |
|  | 39) | Tooth wear status | 4.3±0.8 | 0.60 | 0.80 | 0.19 | 0.50 | 0.78 | 8 | 2 |
| Defective prosthesis | 40) | Unfitness of defective restoration | 4.9±0.3 | 1.00 | 1.00 | 0.06 | 0.00 | 1.00 | 9 | 1 |
|  | 41) | Breakage of defective restoration | 4.9±0.3 | 1.00 | 1.00 | 0.06 | 0.00 | 1.00 | 9 | 1 |
|  | 42) | Unfitness of dentures | 4.9±0.3 | 1.00 | 1.00 | 0.06 | 0.00 | 1.00 | 8 | 2 |
|  | 43) | Denture breakage | 4.9±0.3 | 1.00 | 1.00 | 0.06 | 0.00 | 1.00 | 8 | 2 |
|  | 44) | Denture wearing and removing status | 5.0±0.0 | 1.00 | 1.00 | 0.00 | 0.00 | 1.00 | 8 | 2 |
| Communication | 45) | Expression ability | - | - | - | - | - | - | 0 | 0 |
|  | 46) | Communication ability | 4.6±0.7 | 0.80 | 0.90 | 0.15 | 0.38 | 0.85 | 1 | 9 |
| 1.3. Daily health |  | Vitality (will to live: interest in things) |  |  |  |  |  |  |  |  |
| Vitality | 47) |  | 4.3±0.7 | 0.80 | 0.90 | 0.16 | 0.50 | 0.75 | 1 | 9 |
| Psychological problems | 48) | Psychological problems (depression, bipolar disorder, social isolation, etc.) | 4.2±0.6 | 0.80 | 0.90 | 0.15 | 0.38 | 0.81 | 1 | 9 |
| Lifestyle habits | 49) | Daily lifestyle habits (eating behaviors, lifestyle behaviors, hobbies, etc.) | 4.4±0.7 | 0.80 | 0.90 | 0.16 | 0.50 | 0.78 | 1 | 9 |
| Dietary habits | 50) | Dietary habits (nutritional intake status, diet, etc.) | 4.8±0.4 | 1.00 | 1.00 | 0.09 | 0.00 | 1.00 | 1 | 9 |
| Family composition | 51) | Family composition (living arrangement, family history) | 4.2±0.8 | 0.60 | 0.80 | 0.19 | 0.50 | 0.75 | 1 | 9 |
| Local environment | 52) | Local environment (local characteristics, health and welfare support system, etc.) | - | - | - | - | - | - | 0 | 0 |
| **Part 2. Implementation stage** |  |  |  |  |  |  |  |  |  |  |
| 2.1. Oral health |  |  |  |  |  |  |  |  |  |  |
|  | 53) | Oral health education for oral care methods that suit the characteristics of the older individual for the prevention of oral diseases | 5.0±0.0 | 1.00 | 1.00 | 0.00 | 0.00 | 1.00 | 8 | 2 |
|  | 54) | Clean the inside of the older individual’s mouth using a sponge brush or gauze swab | 5.0±0.0 | 1.00 | 1.00 | 0.00 | 0.00 | 1.00 | 4 | 6 |
|  | 55) | Rinse the inside of the older individual’s mouth using mouthwash and physiological saline | 4.9±0.3 | 1.00 | 1.00 | 0.06 | 0.00 | 1.00 | 5 | 5 |
|  | 56) | Professional toothbrushing customized for the older individual | 4.9±0.3 | 1.00 | 1.00 | 0.06 | 0.00 | 1.00 | 8 | 2 |
|  | 57) | Treat the older individual’s stomatitis and intraoral wounds | 4.8±0.4 | 1.00 | 1.00 | 0.09 | 0.00 | 1.00 | 8 | 2 |
|  | 58) | Professional dental plaque control (Watanabe) on the older individual | 4.8±0.6 | 0.80 | 0.90 | 0.13 | 0.00 | 1.00 | 10 | 0 |
|  | 59) | Teeth cleaning (scaling, polishing, etc.) on the older individual | 4.7±0.7 | 0.80 | 0.90 | 0.14 | 0.00 | 1.00 | 10 | 0 |
|  | 60) | Fluoride application on the older individual for caries control | 4.8±0.6 | 0.80 | 0.90 | 0.13 | 0.00 | 1.00 | 10 | 0 |
|  | 61) | Denture care (cleaning, storage, oral care after removal) for the older individual | 5.0±0.0 | 1.00 | 1.00 | 0.00 | 0.00 | 1.00 | 5 | 5 |
|  | 62) | Treatment for xerostomia (application of lip balm, oral moisturizer, etc.) on the older individual | 4.6±0.5 | 1.00 | 1.00 | 0.11 | 0.50 | 0.80 | 5 | 5 |
|  | 63) | Intra- and extra-oral massage (including oral calisthenics) on the older individual to prevent xerostomia | 4.6±0.7 | 0.80 | 0.90 | 0.15 | 0.38 | 0.85 | 4 | 6 |
| 2.2. Nutrition and diet |  |  |  |  |  |  |  |  |  |  |
|  | 64) | Eating and swallowing function training to improve the older individual’s oral functions | 4.8±0.4 | 1.00 | 1.00 | 0.09 | 0.00 | 1.00 | 6 | 4 |
|  | 65) | Dietary counseling to maintain the older individual’s nutritional status | 4.4±1.0 | 0.80 | 0.90 | 0.22 | 0.50 | 0.80 | 5 | 5 |
|  | 66) | Dietary assessment to maintain the older individual’s nutritional status | 4.2±0.9 | 0.80 | 0.90 | 0.22 | 0.50 | 0.75 | 6 | 4 |
| 2.3. Others |  |  |  |  |  |  |  |  |  |  |
|  | 67) | Implement body position change for the older individual’s oral health care. | - | - | - | - | - | - | 0 | 0 |
|  | 68) | Implement bed and wheelchair mobility. | - | - | - | - | - | - | 0 | 0 |
|  | 69) | Implement first aid and basic life support in case of emergency situation. | 4.6±0.5 | 1.00 | 1.00 | 0.11 | 0.50 | 0.80 | 2 | 8 |
|  | 70) | Communicate with consideration for the older individual’s systemic diseases and psychological characteristics. | 4.4±0.8 | 0.60 | 0.80 | 0.19 | 0.50 | 0.80 | 2 | 8 |
|  | 71) | Provide oral health care- and promotion-related information (oral health education, how to link to/utilize local healthcare services, etc.) to older individuals/guardians. | 4.9±0.3 | 1.00 | 1.00 | 0.06 | 0.00 | 1.00 | 6 | 4 |
| **Part 3. Evaluation stage** |  |  |  |  |  |  |  |  |  |  |
| Oral health status | 72) | Oral health status before and after implementation: Evaluate BOP | 4.8±0.4 | 1.00 | 1.00 | 0.09 | 0.00 | 1.00 | 10 | 0 |
|  | 73) | Oral health status before and after implementation: Evaluate O’Leary index for remaining teeth | 4.7±0.4 | 1.00 | 1.00 | 0.10 | 0.38 | 0.85 | 10 | 0 |
| Oral health behaviors | 74) | Oral health care behaviors before and after implementation (frequency of toothbrushing, interdental care, etc.) | 4.8±0.6 | 0.80 | 0.90 | 0.13 | 0.00 | 1.00 | 5 | 5 |
| Oral health awareness | 75) | Changes in oral health knowledge and awareness before and after implementation | 4.8±0.4 | 1.00 | 1.00 | 0.09 | 0.00 | 1.00 | 6 | 4 |
| Satisfaction | 76) | Participant’s satisfaction with the program (reuse intention, recommendation intention, program satisfaction) | 4.9±0.3 | 1.00 | 1.00 | 0.06 | 0.00 | 1.00 | 5 | 5 |

^*^CVR: content validity ratio,

^**^CVI: content validity index

^***^CV: coefficient of variation
